# Comparison of vaginal natural orifice transluminal endoscopic surgery and multiport conventional laparoscopy for complete pelvic lymphadenectomy: Insights from a single-surgeon experience

**DOI:** 10.1186/s12905-026-04698-3

**Published:** 2026-07-20

**Authors:** Kemal Gungorduk, Serkan Ustuntas, Emre Biton, Berican Sahin Uyar, Berke Nur Ergu, Furkan Karaoz, Varol Gulseren, Munteha Akbulut, Berfin Akan Isler

**Affiliations:** 1 Faculty of Medicine, Department of Obstetrics and Gynecology, Division of Gynecologic Oncology, Mugla Sitki Kocman University, Mugla, Turkey; 2 Faculty of Medicine, Department of Obstetrics and Gynecology, Mugla Sitki Kocman University, Mugla, Turkey; 3 Division of Gynecologic Oncology, Department of Obstetrics and Gynecology, Faculty of Medicine, Katip Celebi University, Izmir, Turkey

**Keywords:** Vaginal natural orifice transluminal endoscopic surgery, Multiport conventional laparoscopy, Complete pelvic lymph node dissection, Endometrial cancer, Pelvic lymphadenectomy

## Abstract

**Background and Objectives:**

We compared the outcomes of complete pelvic lymph node dissection performed via multiport conventional laparoscopy (MCL) versus vaginal natural orifice transluminal endoscopic surgery (vNOTES) in patients with endometrial cancer in whom sentinel lymph node (SLN) mapping was unsuccessful.

**Methods:**

This retrospective cohort study included 42 patients with grade I or II endometrioid endometrial cancer who underwent hysterectomy with bilateral salpingo-oophorectomy, followed by comprehensive retroperitoneal pelvic lymphadenectomy after failed bilateral SLN mapping. Patients underwent either vNOTES (*n* = 21) or MCL (*n* = 21). Demographic characteristics, operative times, perioperative and postoperative complications, pain scores, and quality of recovery-15 (QoR-15) scores were collected and compared between the two groups.

**Results:**

Demographic and clinical characteristics were comparable between the two groups, with no statistically significant differences. Operative times were significantly longer in the vNOTES group (53.8 ± 7.4 min) compared to the MCL group (46.9 ± 9.2 min; *P* = 0.01), whereas estimated blood loss (17.7 ± 5.6 mL vs. 18.8 ± 5.0 mL; *P* = 0.50) and lymph node yield (32.1 ± 6.6 vs. 35.0 ± 8.1; *P* = 0.21) did not differ significantly. Postoperative pain assessed by visual analogue scale (VAS) was lower in the vNOTES group at 6 h (4.3 ± 1.1 vs. 5.5 ± 1.0; *P* = 0.01), 12 h (3.5 ± 0.8 vs. 4.8 ± 1.1; *P* < 0.001), and 24 h (2.1 ± 0.6 vs. 3.1 ± 0.9; *P* < 0.001). These findings were corroborated by the faces pain scale-revised (FPS-R), which demonstrated significantly lower pain in the vNOTES group at all time points. QoR-15 scores at 48 h were higher in the vNOTES group (109.3 ± 4.7 vs. 104.9 ± 4.1; *P* = 0.002), indicating improved postoperative recovery. The observed mean difference of approximately 4.4 points remained below the reported minimal clinically important difference of 8.0 points for this instrument, indicating that the statistically significant benefit should be interpreted with caution regarding its clinical magnitude.

**Conclusion:**

vNOTES for complete pelvic lymphadenectomy offers reduced postoperative pain, scar-free surgery, and enhanced recovery while maintaining staging adequacy comparable to MCL. Future prospective, multicentre, randomised trials with long-term oncologic follow-up are required to validate these findings.

## Introduction

Minimally invasive surgery (MIS), including laparoscopy and robotic-assisted approaches, has become the standard of care for early-stage endometrial cancer. MIS offers several advantages over traditional open surgery, including reduced blood loss, shorter hospital stays, and faster postoperative recovery [1–3]. Accurate surgical staging is essential, particularly the evaluation of lymph node involvement, which remains a key prognostic factor. Sentinel lymph node (SLN) mapping has largely supplanted complete pelvic lymphadenectomy, offering precise staging while minimising surgical morbidity [4].

A recent advancement in MIS is vaginal natural orifice transluminal endoscopic surgery (vNOTES), which uses the vagina as a natural access route, obviating the need for abdominal incisions. This approach offers the potential for further reductions in surgical invasiveness, decreased postoperative pain, and improved cosmetic outcomes. Although vNOTES has been successfully employed in various benign gynaecological procedures, its application is now expanding to oncologic indications, including endometrial cancer staging [5, 6]. Systematic reviews and case series of vNOTES SLN mapping in endometrial cancer report overall detection rates of approximately 98%, with bilateral detection achieved in roughly 94% of cases [5]-outcomes comparable to, and in some instances exceeding, those reported for conventional laparoscopic and robotic-assisted approaches [5, 6].

According to the Memorial Sloan Kettering Cancer Center algorithm for SLN mapping in endometrial cancer, complete lymphadenectomy on the side of failed mapping is indicated [7]. In contemporary practice, bilateral SLN mapping failure occurs in a minority of cases; however, when it does occur, it mandates complete pelvic lymphadenectomy to ensure oncologic staging completeness. At our institution, during the study period, bilateral SLN mapping failure was observed in approximately 12% of all patients undergoing endometrial cancer staging, and those patients constitute the population studied here. At our institution during the same period, the overall SLN detection rate was 92% and the bilateral SLN detection rate was 88%, with unilateral-only mapping in a further proportion of cases; only patients with completely failed bilateral mapping (approximately 12%) were referred for complete pelvic lymphadenectomy and included in this analysis. Although international guidelines recognise the reliability of conventional laparoscopic pelvic lymphadenectomy, studies on complete pelvic lymphadenectomy via the vNOTES approach remain limited.

The primary research question of this study was whether vNOTES offers advantages in postoperative pain and recovery quality compared with MCL for complete pelvic lymphadenectomy, while maintaining equivalent staging adequacy in terms of lymph node yield and perioperative safety. We retrospectively compared perioperative outcomes of complete pelvic lymph node dissection performed using MCL versus vNOTES in patients requiring surgical staging after unsuccessful bilateral SLN mapping.

## Methods

This retrospective cohort study included patients with endometrioid endometrial carcinoma who underwent either vNOTES or MCL hysterectomy with bilateral salpingo-oophorectomy, followed by retroperitoneal complete pelvic lymphadenectomy after unsuccessful bilateral SLN mapping, between January 2023 and November 2025 at Mugla Sitki Kocman University Faculty of Medicine. The study was conducted in accordance with the ethical principles of the 1964 Declaration of Helsinki and was approved retrospectively by the Institutional Review Board (IRB) of Mugla Sitki Kocman University in February 2026 (59/2025–001). Approval was sought retrospectively because the study analysed routinely collected clinical data without additional patient contact; all procedures had been performed as standard care. Written informed consent was obtained from all participants prior to inclusion.

### Patient selection and treatment allocation

Patients were selected according to the following pre-specified criteria: preoperative endometrial biopsy confirming low-grade (grade I or II) endometrioid adenocarcinoma; no preoperative evidence of intraperitoneal, lymphatic, or distant metastases on positron emission tomography/computed tomography, magnetic resonance imaging, or computed tomography; a preoperative recommendation for surgical staging by a multidisciplinary tumour board; and unsuccessful bilateral SLN mapping at the time of surgery.

The choice of surgical approach-vNOTES or MCL-was not randomised. Until mid-2023, all procedures were performed by conventional MCL. From mid-2023 onwards, after the surgeon (K.G.) had completed a dedicated vNOTES training programme and surpassed the institutional learning curve (defined as the first 10 vNOTES cases, in line with published data), vNOTES was offered as the preferred approach to eligible patients, provided they lacked contraindications such as severe vaginal atrophy, insufficient vaginal capacity, or anatomy precluding safe transvaginal access. Patients who declined vNOTES continued to undergo MCL. This temporal introduction of vNOTES represents a potential source of temporal bias, which is acknowledged as a limitation. No differences in patient characteristics between the two groups were observed that would suggest systematic selection bias based on BMI, comorbidity, or prior abdominal surgery (Table 2).

### Surgical technique

All procedures were performed by a single experienced gynaecologic oncologist (K.G.) following standardised techniques. Institutional Enhanced Recovery After Surgery (ERAS) protocols were applied uniformly to both groups (Table 1). Patients received general anaesthesia and were placed in the dorsal lithotomy position.

**Table 1:** Enhanced Recovery After Surgery (ERAS) protocol

Measure	Details
Patient education	Patient counselled regarding surgery, postoperative pain, and other complications to reduce preoperative anxiety. Advised on cessation of smoking and alcohol consumption. Anaemia correction (if necessary); correction of hypoalbuminaemia; information regarding the ERAS programme; written and oral informed consent; preoperative low-fibre diet (starting 3 days before surgery)
Bowel preparation	No mechanical bowel preparation
Preoperative diet	Normal meal allowed up to 6 h before surgery; clear fluids up to 2 h before surgery; carbohydrate-rich fluid (5 mL/kg) administered to patients without diabetes 2 h before surgery
Preoperative medications	Pre-emptive analgesia with paracetamol (650 mg orally, once, 60–75 min before surgery); low-molecular-weight heparin 12 h before surgery
Antibiotic prophylaxis	Ceftriaxone 1 g intravenously 30 min before incision; metronidazole 500 mg intravenously 60 min before incision. Additional doses if surgery exceeded 3 h and/or blood loss exceeded 1,500 mL. Chlorhexidine alcohol for skin cleansing
Nasogastric tube	Not used
Anaesthesia	Total intravenous anaesthesia: fentanyl (0.5–1 mcg/kg), lidocaine (20–30 mg), propofol (1–1.5 mg/kg); non-depolarising neuromuscular blockade; goal-directed fluid therapy; normothermia maintained; intraoperative glucose and blood pressure control; low-tidal-volume, high-frequency ventilation; alveolar recruitment manoeuvres (30 cmH2 for 30 s every 30–60 min). Paracetamol (10 mg/kg) and tramadol (1 mg/kg) administered intravenously during desufflation
Antiemetic prophylaxis	Dexamethasone 4 mg intravenously at induction; ondansetron 4 mg intravenously at the end of surgery
Postoperative analgesia	Paracetamol 1 g intravenously every 6 h for 24 h. Rescue analgesia: diclofenac 75 mg intravenously every 8 h if VAS > 4 (max 3 doses); tramadol 100 mg intravenously if VAS > 6 (max 3 doses)
Fluid therapy	1 mL/kg/h; discontinued at 6 h postoperatively. Fluid bolus of 500 mL if urine output < 30 mL/h
Diet reintroduction	Semi-solid diet within 6 h; solid diet within 12 h after surgery
Postoperative mobilisation	Out of bed for > 4 h on the evening of surgery, including > = 4 walks; sitting in a chair for all meals until discharge
VTE prophylaxis	Low-molecular-weight heparin from postoperative day 1 for 28 days; sequential compression devices while in hospital bed
Urinary catheter	Removed as soon as the patient could walk to the restroom, except in cases of bladder injury
Discharge criteria	Pain well controlled on oral medication; no signs of infection; tolerating oral feeding; independent voiding; walking freely. Follow-up at 1 and 2 weeks after discharge

*VAS* visual analogue scale

For the vNOTES approach, indocyanine green was injected cervically prior to SLN mapping. A 2–3 cm bilateral lateral vaginal fornix incision was made at the 3–5 o'clock and 7–9 o'clock positions to access the retroperitoneal pelvic space bilaterally. This bilateral incision technique-rather than a single posterior colpotomy-was deliberately adopted to facilitate direct, symmetric retroperitoneal access for pelvic lymphadenectomy, representing a modification specific to the retroperitoneal approach described herein. A 7 cm GelPoint V-Path Transvaginal Access Platform (Applied Medical, Rancho Santa Margarita, CA, USA) was used, with CO2 insufflation maintaining retroperitoneal pressure at 8 mmHg. Three trocars were used: a 10 mm 30 degree rigid camera and two 5 mm working ports accommodating bipolar graspers and vessel-sealing devices. Dissection emphasised meticulous identification of the ureter, iliac vessel bifurcations, internal and external iliac vessels, and the obturator nerve. After failed bilateral SLN mapping, a type II pelvic lymphadenectomy was performed on both sides [8].

For the MCL group, a vertical incision above the umbilicus accommodated a 10–12 mm trocar. Pneumoperitoneum was established at 12 mmHg. Three additional 5 mm trocars were inserted under visual guidance. Patients were placed in a 30 degree Trendelenburg tilt to optimise exposure. Pelvic lymphadenectomy was performed according to internationally recognised recommendations [9].

The SLN mapping protocol utilized either methylene blue dye or indocyanine green (ICG), each prepared at a concentration of 1 mg/mL with a total volume of 4 mL. The tracer was administered via cervical stromal injection at the 3 and 9 o'clock positions, combining both superficial (1–4 mm) and deep (1 cm) injections to optimize lymphatic uptake and sentinel lymph node detection. Bilateral SLN mapping failure was defined as the inability to identify any SLN on either side after thorough exploration following dye injection, prompting complete pelvic lymphadenectomy bilaterally per the Memorial Sloan Kettering Cancer Center algorithm.

### Postoperative assessments

Perioperative data were retrospectively extracted from patient charts and electronic hospital records. Variables included demographics (age, BMI, obstetric history, prior surgery), surgical details (procedure type, operative time), and outcomes (pain scores, complications, estimated blood loss, haemoglobin changes within 24 h of surgery, and length of hospital stay).

Postoperative pain was assessed by a trained nurse at 6, 12, and 24 h postoperatively using the visual analogue scale (VAS; 0–10) and the faces pain scale-revised (FPS-R). The quality of recovery-15 (QoR-15) questionnaire-a validated, 15-item instrument with an established minimal clinically important difference (MCID) of 8.0 points in the general surgical population [10], and validated in a Turkish-speaking population [21], was administered at 48 h postoperatively. Scar assessment was performed at the 2-month follow-up visit using the patient and observer scar assessment scale (POSAS) [11]. As vNOTES leaves no abdominal scars, OSAS and PSAS were not applicable in the vNOTES group and are reported only for the MCL group.

### Statistical analysis

Statistical analyses were performed using MedCalc software (v14.0; MedCalc Software Ltd, Mariakerke, Belgium). Categorical variables, expressed as n (%), were compared using the chi-square test or Fisher's exact test. Continuous variables, expressed as mean ± standard deviation (SD), were compared using the independent-samples t-test. All tests were two-sided, with statistical significance set at P < 0.05. No formal sample size calculation was performed prior to data collection, as this was a retrospective analysis. Accordingly, the study was not formally powered for any specific endpoint, and the absence of statistically significant differences in secondary outcomes should not be interpreted as evidence of equivalence. The multiple comparisons performed in this analysis were exploratory in nature; no adjustment for multiplicity was applied, and findings should be interpreted with this limitation in mind.

## Results

**Fig. 1:**
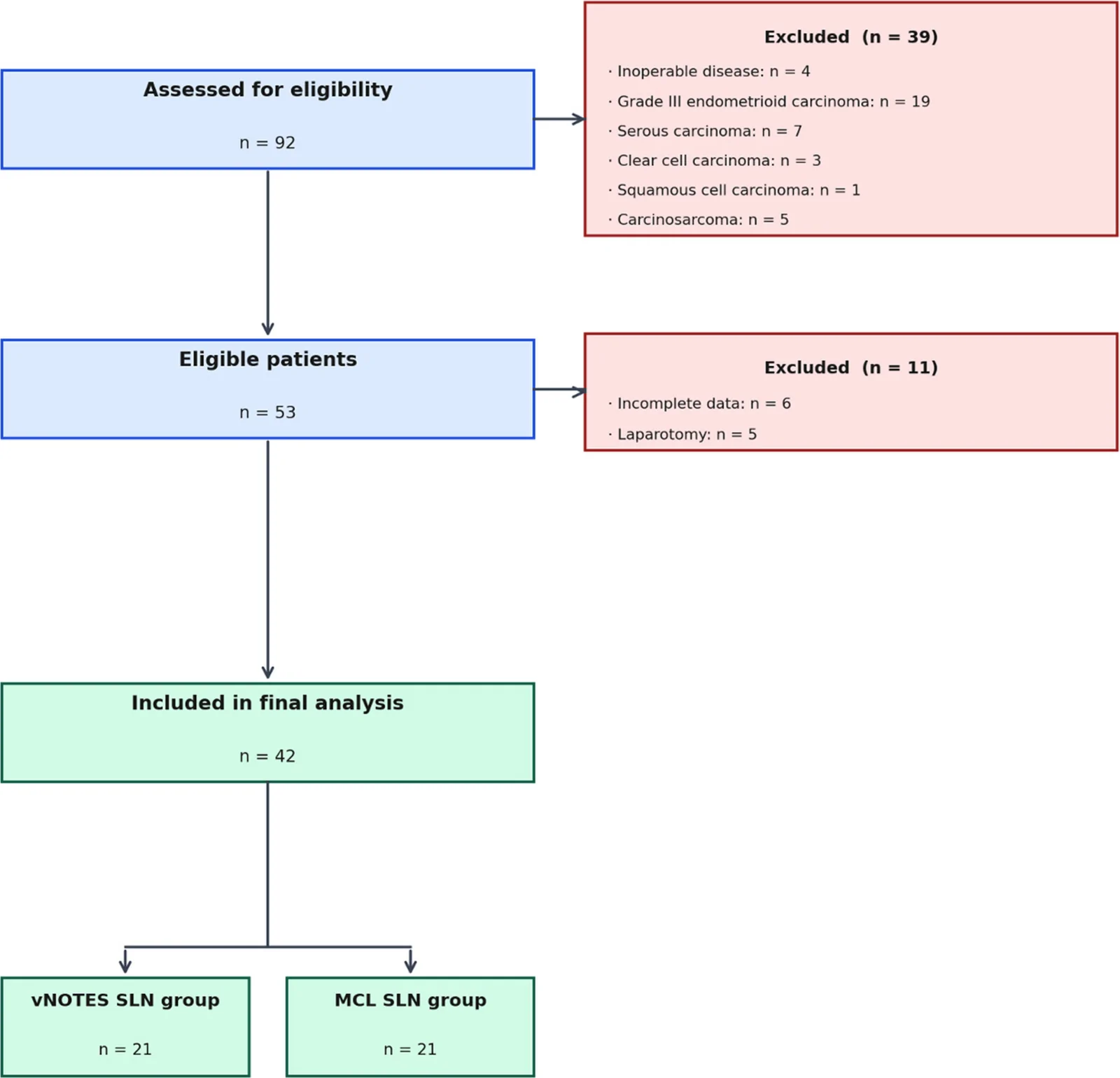
CONSORT-style flow diagram illustrating patient selection. vNOTES SLN, vaginal natural orifice transluminal endoscopic surgery sentinel lymph node mapping; MCL SLN, multiport conventional laparoscopy sentinel lymph node mapping

In total, 42 patients were included: 21 in the vNOTES group and 21 in the MCL group. A CONSORT-style flow diagram illustrating patient identification, exclusions, and final group allocation is presented in Fig. 1. Baseline demographic and clinical characteristics were comparable between the two groups, with no statistically significant differences (Table 2). Mean age was 52.5 ± 12.1 years in the vNOTES group and 55.0 ± 8.4 years in the MCL group (*P* = 0.48). Obesity (BMI ≥ 30 kg/m2) was present in 71.4% and 61.9% of patients in the vNOTES and MCL groups, respectively (*P* = 0.37).

**Table 2:** Demographic and clinical characteristics of patients

Characteristics	vNOTES Group (*n* = 21)	MCL Group (*n* = 21)	*P* Value
Age,a years	52.5 ± 12.1	55.0 ± 8.4	0.48
BMI,a kg/m2	32.4 ± 5.3	30.9 ± 4.4	0.30
BMI > = 30 kg/m2	15 (71.4)	13 (61.9)	0.37
CCI,b			0.68
0	14 (66.7)	15 (71.4)	
I	6 (28.6)	4 (19.0)	
II	1 (4.8)	2 (9.5)	
ASA,b			0.29
I	2 (9.5)	4 (19.0)	
II	5 (23.8)	8 (38.1)	
III	14 (66.7)	9 (42.9)	
Gravida,a	1.6 ± 0.6	1.8 ± 0.6	0.42
Smoking history,b	-	1 (4.8)	1.00
Alcohol consumption,b	6 (28.6)	5 (23.8)	1.00
Previous abdominal surgery,b	10 (47.6)	12 (57.1)	0.75
Caesarean section	6 (60.0)	5 (41.7)	
Myomectomy	-	1 (8.3)	
Appendectomy	2 (20.0)	2 (16.7)	
Cholecystectomy	1 (10.0)	2 (16.7)	
Others	1 (10.0)	2 (16.7)	
Indication for surgery,b			0.58
Grade I endometrioid adenocarcinoma	13 (61.9)	11 (52.4)	
Grade II endometrioid adenocarcinoma	8 (38.1)	10 (47.6)	

*BMI* body mass index, *CCI* Charlson Comorbidity Index, *ASA* American Society of Anesthesiologists, *MCL* multiport conventional laparoscopy. a Mean ± SD; b n (%). cThe SD for age in the MCL group has been corrected from the originally submitted ± 1.7 to ± 8.4, reflecting the true data distribution

Operative time was significantly longer in the vNOTES group (53.8 ± 7.4 min) compared with the MCL group (46.9 ± 9.2 min; *P* = 0.01) (Table 3). Estimated blood loss and lymph node yield did not differ significantly between groups. Pain intensity assessed by VAS was consistently lower in the vNOTES group at all postoperative time points: 6 h (4.3 ± 1.1 vs. 5.5 ± 1.0; *P* = 0.01), 12 h (3.5 ± 0.8 vs. 4.8 ± 1.1; P < 0.001), and 24 h (2.1 ± 0.6 vs. 3.1 ± 0.9; P < 0.001). FPS-R scores were similarly lower in the vNOTES group at all time points. QoR-15 total score at 48 h was significantly higher in the vNOTES group (109.3 ± 4.7 vs. 104.9 ± 4.1; *P* = 0.002), with the observed mean difference of approximately 4.4 points falling below the published MCID of 8.0 points for the general surgical population, suggesting that although the difference is statistically significant, its clinical magnitude warrants cautious interpretation. Significant improvements were also observed in Part A (physical comfort and support; *P* = 0.01) and Part B (emotional well-being; *P* = 0.007). Postoperative hospital stay was comparable between the two groups (1.4 ± 0.5 vs. 1.5 ± 0.6 days; *P* = 0.52). OSAS and PSAS scar scores were assessed only in the MCL group (18.4 ± 2.7 and 18.5 ± 2.2, respectively); these instruments were not applicable in the vNOTES group given the absence of abdominal incisions.

**Table 3:** Study outcomes

Characteristics	vNOTES Group (*n* = 21)	MCL Group (*n* = 21)	*P* Value
Operative time,a min	53.8 ± 7.4	46.9 ± 9.2	0.01
Number of lymph nodes retrieved,a	32.1 ± 6.6	35.0 ± 8.1	0.21
Estimated blood loss,a mL	17.7 ± 5.6	18.8 ± 5.0	0.50
VAS pain score,a			
6 h postoperatively	4.3 ± 1.1	5.5 ± 1.0	0.01
12 h postoperatively	3.5 ± 0.8	4.8 ± 1.1	< 0.001
24 h postoperatively	2.1 ± 0.6	3.1 ± 0.9	< 0.001
FPS-R pain score,a			
6 h postoperatively	4.9 ± 1.2	6.2 ± 1.4	0.002
12 h postoperatively	3.1 ± 1.1	4.6 ± 0.9	< 0.001
24 h postoperatively	2.2 ± 0.7	2.8 ± 1.0	0.04
Additional analgesic requirement,b	3 (14.3)	5 (23.8)	0.69
Blood transfusion,b	-	-	-
Intraoperative complications,b	-	-	-
Postoperative complications,b	1 (4.8)	1 (4.8)	1.00
Lymphocele (no intervention required)	-	1 (4.8)	
Lymphocele requiring percutaneous drainage	1 (4.8)	-	
QoR-15 score at 48 h,a			
Part A (physical comfort and support)	75.1 ± 4.0	71.9 ± 3.6	0.01
Part B (emotional well-being)	34.8 ± 1.9	32.9 ± 2.3	0.007
Total	109.3 ± 4.7	104.9 ± 4.1	0.002
Postoperative hospital stay,a days	1.4 ± 0.5	1.5 ± 0.6	0.52
OSAS score (2-month follow-up),a	N/A (scar-free)	18.4 ± 2.7	-
PSAS score (2-month follow-up),a	N/A (scar-free)	18.5 ± 2.2	-

*VAS* visual analogue scale, *FPS-R* faces pain scale-revised, *QoR-15* quality of recovery-15 questionnaire, *OSAS* observer scar assessment scale, *PSAS* patient scar assessment scale, *MCL* multiport conventional laparoscopy, *N/A* not applicable. a Mean ± SD; b n (%)

## Discussion

To our knowledge, this study represents the first single-surgeon comparative analysis of vNOTES versus MCL for performing complete pelvic lymphadenectomy during the surgical staging of endometrial cancer. Our findings indicate that vNOTES was associated with meaningful perioperative and patient-centred advantages-including reduced postoperative pain and improved short-term recovery quality-while maintaining staging adequacy comparable to MCL in terms of lymph node yield and perioperative safety.

Postoperative pain is a critical outcome metric in minimally invasive oncologic surgery, as lower pain levels facilitate earlier mobilisation and contribute to enhanced recovery. The primary driver of reduced pain in the vNOTES group is the complete absence of abdominal wall incisions. The vNOTES approach eliminates trocar-site trauma, reduces the risk of somatic nerve injury, and mitigates the inflammatory response triggered by disruption of the abdominal fascia and peritoneum. Previous studies have demonstrated a direct relationship between postoperative pain and both the number and calibre of laparoscopic port sites [12–15]. By utilising vaginal access exclusively, vNOTES removes this source of postoperative nociception. Furthermore, the use of lower insufflation pressures (8–10 mmHg versus 12–15 mmHg in conventional laparoscopy) reduces peritoneal irritation and decreases referred shoulder pain-a frequent and distressing complication of traditional laparoscopy [12–15].

Beyond its analgesic advantages, vNOTES eliminates risks directly attributable to abdominal access, including incisional hernia (reported in 0.5–2% of laparoscopic procedures), trocar-site infection, and port-site metastasis [16, 17].

Although statistically significant, the observed difference in QoR-15 total score between groups (approximately 4.4 points) fell below the published MCID of 8.0 points for the general surgical population. This finding should be interpreted cautiously: a statistically significant result in a small sample does not necessarily translate into a clinically meaningful difference. It is possible that in a larger cohort, or in patients with more complex comorbidities, the clinical significance of this difference would be greater. Future studies adequately powered for the QoR-15 MCID as the primary endpoint are required.

The enhanced recovery profile of vNOTES was further supported by the significantly higher QoR-15 subscale scores in both Part A (physical comfort and support) and Part B (emotional well-being). However, postoperative hospital stay was comparable between groups, suggesting that the improved pain control and recovery quality did not translate into earlier discharge in this cohort.

A clinically important advantage of retroperitoneal vNOTES lymphadenectomy is its capacity to enable comprehensive surgical staging with minimal reliance on steep Trendelenburg positioning [15, 18]. This is particularly relevant in patients with obesity and cardiopulmonary comorbidities. In our cohort, 71.4% of vNOTES and 61.9% of MCL patients had a BMI > = 30 kg/m2. Prolonged steep Trendelenburg positioning introduces haemodynamic and respiratory challenges including reduced lung compliance, increased airway pressure, decreased functional residual capacity, elevated intracranial pressure, and haemodynamic instability [19].

Regarding staging adequacy, lymph node yields were comparable between approaches (32.1 ± 6.6 for vNOTES vs. 35.0 ± 8.1 for MCL; *P* = 0.21), suggesting that transvaginal retroperitoneal access achieves equivalent staging completeness to conventional laparoscopy. Wong et al. reported a median lymph node yield of 11.5 in 13 patients undergoing pelvic lymph node dissection via vNOTES [15], while Matak et al. demonstrated accurate nodal identification and removal via retroperitoneal vNOTES, including in obese patients [20]. The complication rates were low and comparable between groups. However, we emphasise that lymph node yield and short-term complication data represent surrogate measures of staging adequacy; they are not oncologic outcomes per se. Recurrence-free survival and overall survival-the true measures of oncologic adequacy-were not assessable within the short follow-up period of this study.

The longer operative time observed in the vNOTES group (53.8 vs. 46.9 min; *P* = 0.01) most likely reflects the inherent learning curve associated with the transvaginal retroperitoneal approach. Our institutional threshold of approximately 10 cases to reach procedural proficiency is consistent with published learning curve analyses for vNOTES procedures [12]. It is anticipated that operative times will converge with increasing surgeon experience. Additionally, vNOTES is not appropriate for all patients; contraindications include severe vaginal atrophy, nulliparity with limited vaginal capacity, and cases requiring extensive para-aortic dissection.

Regarding clinical practice implications, vNOTES pelvic lymphadenectomy may be preferred in patients at high risk from abdominal wall access complications (obesity, previous laparotomy, or immunosuppression), in those with significant cardiopulmonary comorbidities that make prolonged Trendelenburg positioning hazardous, and in patients who place high value on cosmetic outcomes. However, until randomised controlled trial data are available, vNOTES should be considered complementary to, rather than a replacement for, established MCL in this indication.

### Limitations

This study has several limitations. First, the retrospective design introduces the potential for residual confounding and selection bias. The sequential introduction of vNOTES during the study period may have produced temporal bias, as patient management and institutional experience evolved over time. Second, the small sample size (*n* = 21 per group) limits statistical power and generalisability; no formal power calculation was performed, and the risk of Type II error for secondary endpoints is acknowledged. Third, the single-surgeon, single-centre design minimises operator variability but limits external validity. Fourth, multiple statistical comparisons were performed without adjustment for multiplicity; all findings should therefore be considered exploratory. Fifth, pain assessment was performed by trained nursing staff who were not blinded to the surgical approach, introducing the possibility of observer bias. Sixth, and most importantly, the follow-up period was limited to 48 h for QoR-15 and 2 months for scar assessment; consequently, no data on long-term oncologic outcomes-including recurrence-free survival and overall survival-are available. These data are essential for definitively evaluating the oncologic safety of vNOTES pelvic lymphadenectomy. Finally, whether the ERAS protocol was applied with identical fidelity in both groups across the entire study period cannot be fully verified from retrospective records.

## Conclusion

vNOTES for complete pelvic lymphadenectomy provides a clinically meaningful combination of reduced postoperative pain, scarless surgery, minimal reliance on Trendelenburg positioning, and improved short-term recovery quality, while maintaining staging adequacy-as measured by lymph node yield and perioperative safety-comparable to MCL. The observed QoR-15 benefit, although statistically significant, should be interpreted in the context of the small sample size and the MCID threshold. Future prospective, randomised, multicentre trials incorporating long-term oncologic outcome data are required to validate these findings.

## Data Availability

All data generated or analyzed during this study are included in this published article.

## Abbreviations

vNOTES: Vaginal natural orifice transluminal endoscopic surgery
MCL: Multiport conventional laparoscopy
SLN: Sentinel lymph node
QoR-15: Quality of recovery-15
MIS: Minimally invasive surgery
IRB: Institutional Review Board
BMI: Body mass index
VAS: Visual analogue scale
FPS-R: Faces pain scale-revised
OSAS: Observer scar assessment scale
PSAS: Patient scar assessment scale
ERAS: Enhanced recovery after surgery
MCID: Minimal clinically important difference
